# Do the American guideline-based leisure time physical activity levels for civilians benefit the mental health of military personnel?

**DOI:** 10.3389/fpsyt.2023.1255516

**Published:** 2023-11-14

**Authors:** Kun-Zhe Tsai, Pang-Yen Liu, Yen-Po Lin, Chen-Chih Chu, Wei-Chun Huang, Xuemei Sui, Carl J. Lavie, Gen-Min Lin

**Affiliations:** ^1^Department of Medicine, Hualien Armed Forces General Hospital, Hualien, Taiwan; ^2^Department of Stomatology of Periodontology, Mackay Memorial Hospital, Taipei, Taiwan; ^3^Department of Dentistry, Tri-Service General Hospital, National Defense Medical Center, Taipei, Taiwan; ^4^Department of Medicine, Tri-Service General Hospital, National Defense Medical Center, Taipei, Taiwan; ^5^Department of Critical Care Medicine, Taipei Tzu Chi Hospital, New Taipei City, Taiwan; ^6^Department of Critical Care Medicine, Kaohsiung Veterans General Hospital, Kaohsiung, Taiwan; ^7^Department of Exercise Science, Arnold School of Public Health, University of South Carolina, Columbia, SC, United States; ^8^Ochsner Clinical School, John Ochsner Heart and Vascular Institute, The University of Queensland School of Medicine, New Orleans, LA, United States

**Keywords:** mental health, psychological distress, suicide ideation, leisure time physical activity, military personnel

## Abstract

**Backgrounds:**

This study aimed to clarify the association of American guideline-based leisure time physical activity (PA) level with mental health in 4,080 military personnel in Taiwan.

**Methods:**

The moderate intensity PA level was assessed according to the total running time per week (wk) reported in a self-administered questionnaire over the previous 6  months and was categorized into PA level <150, 150–299, and ≥300  min/wk. Mental stress was assessed by the Brief Symptom Rating Scale (BSRS)-5 for which ≥15 points were classified as great mental stress. Suicide ideation (SI) was graded as 1 for mild, 2 for moderate, and 3 or 4 for severe. Multivariable logistic regression analysis was employed to determine the association between PA and mental health while adjusting for demographics, smoking, alcohol intake, betel nut chewing, and obesity.

**Results:**

As compared to participants with a PA level of <150  min/wk., those with PA levels 150–299  min/wk. and ≥  300  min/wk. had a lower possibility of SI ≥1 [odds ratios (ORs) and 95% confidence intervals (CIs): 0.58 (0.40–0.83) and 0.23 (0.14–0.36), respectively] and SI ≥1 and/or BSRS-5 ≥  15 [ORs: 0.55 (0.39–0.79) and 0.21 (0.13–0.34), respectively]. The possibilities were more significantly lower for SI ≥2 [ORs: 0.37 (0.20–0.68) and 0.10 (0.04–0.26), respectively] and SI ≥2 and/or BSRS-5 ≥  15 [ORs: 0.35 (0.20–0.62) and 0.10 (0.04–0.25), respectively].

**Conclusion:**

Our findings indicate that participating in moderate-intensity leisure time PA level for ≥150  min/wk. may have a positive effect on mental health among military personnel. The impact appears to be even more significant when engaging in a higher PA level of ≥300  min/wk.

## Introduction

Mental health disorder, manifesting as psychological distress, is a leading cause of the global health burden (1). One in eight individuals worldwide encounters a mental health disorder, and nearly half of all individuals experience such a disorder at some point in their life (1). For military personnel, exposure to combat and rescue, witnessing traumatic events, and the military culture, characterized by strict hierarchies, long deployments, and a sense of duty, can lead to a range of mental health issues, e.g., suicidality, insomnia, anxiety, hostility, depression, and interpersonal sensitivity (2). Suicidality is a serious issue that has not declined appreciably in several decades, and there is no accurate predictor of increased risk for suicide (3). Previous reports have shown that nearly 24 out of every 100,000 soldiers in the US died by suicide (4, 5). Although several studies have been done in the armed forces of the US in the past decade and recently in 2020–21, when the suicide rate in the armed forces reduced approximately 15%, the suicide rate again increased rapidly in the first quarter of 2023 (6, 7). With this alarming trend, mental health promotion has been a critical agenda for the armed forces.

Physical activity (PA) forms an integral part of military life because physical training is designed to improve physical fitness and is always encouraged and even required. The newly published World Health Organization (WHO) guidelines report that frequent PA has a beneficial impact on cardiometabolic as well as cardiovascular disease (CVD) risk, and is also therapeutic for people with psychological distress (8). The effect of PA is even comparable to that of anti-depressants and psychotherapy in some cases (9). We have shown very marked improvements in psychological distress, including depression, anxiety, and hostility, in patients with CVD following a formal PA/cardiac rehabilitation program that improves levels of cardiorespiratory fitness (CRF) (10–15). Despite growing recognition of the importance of frequent PA to improve mental health (8), these investigations did not provide a specific threshold. The American guideline recommends a moderate-intensity leisure time PA level, e.g., running ≥150 min/week (wk) for civilian adults (8, 16). However, it is unclear whether the guideline-based PA level also benefits mental health for military personnel. Therefore, this study aimed to examine the association between moderate-intensity PA levels during leisure time and mental health in a large sample of military subjects in Taiwan.

## Materials and methods

### Study population

Individuals for this cross-sectional study were included from the main study of the cardiorespiratory fitness and health in the armed forces (CHIEF) in Taiwan (17). All participants underwent regular annual whole-body health examinations in the Hualien Armed Forced General Hospital, the only military referral hospital in East Taiwan in 2014. Only the participants who completed the available questionnaire on leisure time PA levels and psychological distress assessment were recruited for this study (18, 19). The Institutional Ethics Committee approval (No. 16-05-008) from the Mennonite Christian Hospital in Taiwan was obtained at the beginning of the study, and all participants gave informed consent.

### Clinical and biochemical assessment

Each participant responded to a questionnaire regarding their personal information and substance use experience (cigarette smoking, alcohol drinking, and betel nut chewing). Anthropometric parameters for waist circumference, body height, and body weight were further measured in a standing position. The body mass index (BMI) was calculated as body weight (kilograms) divided by the square of body height (meters). General obesity was defined as BMI ≥27 kg/m^2^, and abdominal obesity was defined as a waist circumference ≥90 cm in men and ≥80 cm in women (20–22).

The hemodynamic information about systolic blood pressure (SBP), diastolic blood pressure (DBP), and pulse rate was obtained from the right upper arm, using the automatic blood pressure monitoring machine (Parama-Tech Co Ltd., Fukuoka, Japan). Serum metabolic biomarkers, including total cholesterol, low-density lipoprotein cholesterol (LDL-C), high-density lipoprotein cholesterol (HDL-C), triglyceride, fasting glucose (FPG), serum uric acid (SUA), blood urea nitrogen (BUN), and creatinine, and blood cells information about white blood cell count (WBC), red blood cell count (RBC), hemoglobin, and platelet count were obtained from participants’ overnight fasting blood samples (17, 23).

### Mental health assessment

Mental health was assessed using one question on suicide ideation (SI) and the 5-item Brief Symptom Rating Scale (BSRS-5) scores (24–26), which comprised trouble falling asleep (insomnia), feeling tense (anxiety), feeling easily annoyed or irritated (hostility), feeling low in mood (depression), and feeling inferior to others (interpersonal sensitivity). The scoring of each domain of the BSRS-5 and SI used a five-point Likert-type scale, where 0 = not at all, 1 = mild, 2 = moderate, 3 = severe, and 4 = extremely severe. Traditionally, normal, slight, moderate, and great psychological distress has been defined by the sum of BSRS-5 scores ≤5, 6–9, 10–14, and ≥ 15, respectively. Internal consistency (Cronbach alpha) coefficients for the BSRS-5 score ranged between 0.77 and 0.90. The test–retest reliability coefficient was 0.82 (24). In this study, those with SI ≥2 and the BSRS-5 ≥ 15 were considered to have great psychological distress, which was the main outcome in this study. The secondary outcomes in this study were the psychological distress separately assessed by various degrees of SI, the BSRS-5 scores >5, ≥10, and ≥ 15, and each domain of the BSRS-5 ≥ 2 (over moderate degree).

### Moderate intensity PA level

Through a questionnaire, the moderate-intensity PA level of each participant was assessed by weekly total running time on average during leisure time in the past 6 months based on the 2018 American guideline, with <150, 150–299, and ≥300 min/wk. considered as low, adequate and high levels, respectively (16).

### Statistical analysis

The demographic and clinical characteristics were expressed as numbers (%) for categorical data, and mean ± standard deviation (SD) for continuous data. The chi-square test was used to compare categorical variables, and the analysis of variance (ANOVA) test was used to compare continuous variables between those with and without psychological distress. Multiple logistic regression analysis was used to determine the odds ratio (OR) and 95% confidence interval (CI) of psychological distress. We conducted two stages of analysis. In the first stage, the univariate analysis model, only the guideline-based PA levels were entered as a predictor. In the second stage, the multinomial analysis model, we entered potential confounders in the regression analysis and then tested if there was a significant change in the association. Age was treated as a continuous variable, and sex, specialty, cigarette smoking, alcohol drinking, betel nut chewing, and general and abdominal obesity were treated as categorical variables. The possibility of any suicide ideation (≥1) with and without BSRS-5 scores >5, ≥10, and ≥15 was, respectively, analyzed, and the possibility of moderate or more severe (≥2) suicide ideation with and without BSRS-5 scores >5, ≥10, and ≥15 was also analyzed. The software SPSS 26.0 (IBM Corp., Armonk, NY, United States) was used to establish a database and for subsequent statistical analysis. A two-sided *p*-value of less than 0.05 was considered to indicate statistical significance.

## Results

In total, 4,080 individuals with complete data on leisure time PA levels, as well as mental health information, were included in this study for analysis. The demographics and characteristics of the study population are shown in Table 1. Their average age was approximately 29.2 years, and 89.9% of them were males. A higher prevalence of alcohol consumption and betel nut chewing was observed in those with any suicide ideation or BSRS ≥15. Furthermore, those without SI and BSRS <15 had higher PA levels.

**Table 1:** Characteristics of the study participants.

	ALL (*n* = 4,080)	Without suicidal ideation and BSRS <15 (*n* = 4,021)	With suicidal ideation ≥2 or BSRS ≥15 (*n* = 59)	*p* value
**Moderate intensity PA level**				
<150 min/wk	895 (21.9)	862 (21.4)	33 (55.9)	
150–299 min/wk	1,554 (38.1)	1,534 (38.1)	20 (33.9)	<0.001
≥300 min/wk	1,631 (40.0)	1,625 (40.4)	6 (10.2)	
Age, mean (SD), years	29.22 (5.95)	29.23 (5.95)	28.24 (5.61)	0.20
Male sex	3,669 (89.9)	3,618 (90.0)	51 (86.4)	0.37
**Specialty**				
Army	2,107 (51.6)	2069 (51.5)	38 (64.4)	
Navy	849 (20.8)	838 (20.8)	11 (18.6)	0.10
Air force	1,124 (27.5)	1,114 (27.7)	10 (16.9)	
**Unhealthy behavior**				
Cigarette smoking	1,426 (35.0)	1,400 (34.8)	26 (44.1)	0.13
Alcohol drinking	1,686 (41.3)	1,654 (41.4)	32 (54.2)	0.04
Betel nut chewing	439 (10.8)	428 (10.6)	11 (18.6)	0.04
SBP, mean (SD), mmHg	117.18 (13.51)	117.20 (13.53)	116.19 (11.98)	0.56
DBP, mean (SD), mmHg	70.03 (10.14)	70.05 (10.16)	68.42 (9.32)	0.22
Plus rate, mean (SD), bpm	72.51 (10.79)	72.51 (10.78)	72.68 (11.39)	0.90
BMI, mean (SD), kg/m^2^	24.63 (3.15)	24.63 (3.16)	24.47 (2.88)	0.69
Waist circumference, mean (SD), cm	82.37 (8.46)	82.37 (8.47)	82.30 (7.72)	0.94
**Obesity**				
General obesity	860 (21.1)	850 (21.1)	10 (16.9)	0.43
Abdominal obesity	939 (23.0)	925 (23.0)	14 (23.7)	0.89
**Blood test, mean (SD)**				
Total cholesterol, mg/dL	173.70 (33.64)	173.71 (33.58)	172.44 (37.94)	0.77
LDL-C, mg/dL	104.70 (29.50)	104.75 (29.50)	101.37 (29.44)	0.38
HDL-C, mg/dL	48.77 (10.27)	48.76 (10.26)	49.10 (11.01)	0.80
Triglyceride, mg/dL	111.30 (96.56)	111.08 (94.01)	126.36 (207.27)	0.22
FPG, mg/dL	93.15 (13.03)	93.17 (13.09)	91.98 (7.80)	0.48
SUA, mg/dL	6.53 (1.40)	6.53 (1.40)	6.50 (1.30)	0.90
BUN, mg/dL	12.68 (2.87)	12.69 (2.87)	12.33 (2.91)	0.33
Creatinine, mg/dL	0.93 (0.13)	0.93 (0.13)	0.92 (0.14)	0.38
GFR, ml/min/1.73m^2^	100.72 (14.92)	100.68 (14.81)	103.48 (21.52)	0.15
AST, U/L	20.29 (8.66)	20.28 (8.62)	20.95 (10.80)	0.55
ALT, U/L	22.01 (17.19)	22.00 (17.17)	22.73 (18.32)	0.74
WBC, 10^3^/uL	6.73 (1.70)	6.73 (1.70)	6.52 (1.72)	0.34
RBC, 10^3^/uL	5.27 (0.49)	5.27 (0.49)	5.30 (0.54)	0.59
Hemoglobin, g/dL	14.95 (1.18)	14.95 (1.18)	15.02 (1.03)	0.64
HCT, %	44.63 (3.09)	44.63 (3.10)	44.75 (2.88)	0.77
Platelet, 10^3^/uL	255.07 (51.21)	254.95 (51.21)	263.39 (50.65)	0.20
MCV, fL	85.10 (6.35)	85.11 (6.34)	84.89 (6.65)	0.79

PA, physical activity; SBP, systolic blood pressure; DBP, diastolic blood pressure; BMI, body mass index; LDL-C, low-density lipoprotein cholesterol; HDL-C, high-density lipoprotein cholesterol; FPG, fasting plasma glucose; SUA, serum uric acid; BUN, blood urea nitrogen; GFR, glomerular filtration rate; AST, aspartate aminotransferase; ALT, alanine aminotransferase; WBC, white blood cell count; RBC, red blood cell count; HCT, hematocrit; MCV, mean corpuscular volume.

The results of multiple logistic regression analysis for the association between guideline-based moderate-intensity PA levels and psychological distress items are shown in Table 2. As compared to those with a PA level of <150 min/wk., those with a PA level of 150–299 min/wk. had lower odds of suffering from moderate or more severe depression [OR and 95% CI: 0.67 (0.49–0.91)] and interpersonal sensitivity [OR: 0.67 (0.47–0.96)]. In addition, those with a PA level of ≥300 min/wk. had lower odds of suffering from moderate or more severe insomnia, anxiety, hostility, depression, and interpersonal sensitivity [ORs: 0.48 (0.36–0.65), 0.31 (0.21–0.45), 0.47 (0.34–0.65), 0.29 (0.20–0.43), and 0.43 (0.29–0.64), respectively]. With regard to the BSRS-5 score (Supplementary Table S1), there was a tendency for those with a PA level of ≥300 min/wk. to have lower odds of BSRS-5 ≥ 15 [OR: 0.21 (0.04–1.04)], BSRS-5 ≥ 10 [OR: 0.38 (0.21–0.69)], and BSRS-5 > 5 [OR: 0.41 (0.31–0.55)], as compared to those with a PA level of <150 min/wk.

**Table 2:** Association of physical activity levels with various moderate to extremely severe psychological distress.

	Model 1 (Unadjusted)									
	Insomnia ≥2 (*n* = 356)		Anxiety ≥2 (*n* = 228)		Hostility ≥2 (*n* = 299)		Depression ≥2 (*n* = 217)		Interpersonal sensitivity ≥2 (*n* = 179)	
	OR (95% CI)	*p* value	OR (95% CI)	*p* value	OR (95% CI)	*p* value	OR (95% CI)	*p* value	OR (95% CI)	*p* value
<150	1.00		1.00		1.00		1.00		1.00	
150–299	0.86 (0.66–1.11)	0.24	0.76 (0.56–1.03)	0.07	0.82 (0.62–1.09)	0.17	0.67 (0.49–0.92)	0.01	0.68 (0.48–0.96)	0.03
≥300	0.47 (0.35–0.63)	<0.001	0.30 (0.20–0.43)	<0.001	0.45 (0.33–0.62)	<0.001	0.28 (0.19–0.41)	<0.001	0.41 (0.28–0.61)	<0.001
	Model 2 (Multivariable-adjusted)*									
<150	1.00		1.00		1.00		1.00		1.00	
150–299	0.82 (0.62–1.07)	0.13	0.74 (0.54–1.01)	0.057	0.81 (0.61–1.08)	0.14	0.67 (0.49–0.91)	0.01	0.67 (0.47–0.96)	0.02
≥300	0.48 (0.36–0.65)	<0.001	0.31 (0.21–0.45)	<0.001	0.47 (0.34–0.65)	<0.001	0.29 (0.20–0.43)	<0.001	0.43 (0.29–0.64)	<0.001

PA, physical activity; OR, odds ratio; CI, confidence interval.

^*^Adjusted for age, sex, specialty, cigarette smoking, alcohol drinking, betel nut chewing, general obesity, and abdominal obesity.

Table 3 reveals the results of multiple logistic regression analysis of American guideline-based moderate-intensity PA levels for SI and/or various grades of psychological distress. For SI ≥1, an inverse dose–response relationship was found between PA levels and SI with psychological distress. As compared to those with a PA level of <150 min/wk., those with a PA level of 150–299 min/wk. and a PA level of ≥300 min/wk. were observed to be less likely to have any SI [ORs: 0.58 (0.40–0.83) and 0.23 (0.14–0.36), respectively], any SI and/or BSRS-5 ≥ 15 [ORs: 0.55 (0.39–0.79) and 0.21 (0.13–0.34), respectively], any SI and/or BSRS-5 ≥ 10 [ORs: 0.63 (0.45–0.87) and 0.27 (0.18–0.41), respectively], and any SI and/or BSRS-5 > 5 [OR: 0.72 (0.57–0.91) and 0.37 (0.29–0.49), respectively]. For moderate or more severe SI (≥2), such an inverse dose–response relationship still existed and became more obvious. As compared to those with a PA level of <150 min/wk., those with a PA level of 150–299 min/wk. and a PA level of ≥300 min/wk. were observed to be less likely to have an SI ≥2 [OR: 0.37 (0.20–0.68) and 0.10 (0.04–0.26), respectively], an SI ≥2 and/or BSRS-5 ≥ 15 [OR: 0.35 (0.20–0.62) and 0.10 (0.04–0.25), respectively], an SI ≥2 and/or BSRS-5 ≥ 10 [OR: 0.52 (0.34–0.79) and 0.24 (0.14–0.40), respectively], and an SI ≥2 and/or BSRS-5 > 5 [OR: 0.69 (0.54–0.89) and 0.36 (0.27–0.48), respectively].

**Table 3:** Association of physical activity levels with suicidal ideation and different grades of psychological distress.

	Model 1 (unadjusted)							
PA levels (min/wk)	Suicide ideation		Suicide ideation and/or BSRS ≥15		Suicide ideation and/or BSRS ≥10		Suicide ideation and/or BSRS >5	
	OR (95% CI)	*p* value	OR (95% CI)	*p* value	OR (95% CI)	*p* value	OR (95% CI)	*p* value
Suicide ideation ≥1	(*n* = 151)		(*n* = 156)		(*n* = 200)		(*n* = 442)	
<150	1.00		1.00		1.00		1.00	
150–299	0.59 (0.41–0.84)	0.004	0.56 (0.39–0.79)	0.001	0.64 (0.47–0.88)	0.007	0.75 (0.59–0.94)	0.01
≥300	0.22 (0.14–0.35)	<0.001	0.21 (0.13–0.33)	<0.001	0.27 (0.18–0.40)	<0.001	0.37 (0.28–0.48)	<0.001
Suicide ideation ≥2	(*n* = 51)		(*n* = 59)		(*n* = 117)		(*n* = 402)	
<150	1.00		1.00		1.00		1.00	
150–299	0.36 (0.20–0.66)	0.001	0.34 (0.19–0.60)	<0.001	0.53 (0.35–0.79)	0.002	0.72 (0.57–0.92)	0.009
≥300	0.10 (0.04–0.25)	<0.001	0.10 (0.04–0.23)	<0.001	0.24 (0.14–0.39)	<0.001	0.35 (0.27–0.47)	<0.001
	Model 2 (Multivariable-adjusted)*							
Suicide ideation ≥1	(*n* = 151)		(*n* = 156)		(*n* = 200)		(*n* = 442)	
<150	1.00		1.00		1.00		1.00	
150–299	0.58 (0.40–0.83)	0.003	0.55 (0.39–0.79)	0.001	0.63 (0.45–0.87)	0.005	0.72 (0.57–0.91)	0.007
≥300	0.23 (0.14–0.36)	<0.001	0.21 (0.13–0.34)	<0.001	0.27 (0.18–0.41)	<0.001	0.37 (0.29–0.49)	<0.001
Suicide ideation ≥2	(*n* = 51)		(*n* = 59)		(*n* = 117)		(*n* = 402)	
<150	1.00		1.00		1.00		1.00	
150–299	0.37 (0.20–0.68)	0.001	0.35 (0.20–0.62)	<0.001	0.52 (0.34–0.79)	0.002	0.69 (0.54–0.89)	0.004
≥300	0.10 (0.04–0.26)	<0.001	0.10 (0.04–0.25)	<0.001	0.24 (0.14–0.40)	<0.001	0.36 (0.27–0.48)	<0.001

PA, physical activity; OR, odds ratio; CI, confidence interval.

^*^Adjusted for age, sex, specialty, cigarette smoking, alcohol drinking, betel nut chewing, general obesity, and abdominal obesity.

## Discussion

Our findings suggested that military personnel engaging in a moderate intensity PA level of >150 min/wk. in leisure time might have lower psychological distress, particularly in depression and interpersonal sensitivity, and be protected from any SI. Moreover, adhering to the highest PA level that the guideline recommended, ≥300 min/wk., may provide additional benefits for mental health in the military.

The impacts of PA on mental health are incredibly extensive and beneficial for physical or mental illness regardless of age stratum. Higher intensity PA levels have been associated with greater improvements in psychological symptoms (1). In addition, aerobic PA is found to be an important protective factor against health-threatening reactions to acute psychosocial stress (27), which can enhance the consolidation and subsequent recall of fear extinction learning (28). Consistent with our findings, a prior study conducted in the Brazilian Army highlighted the dose–response association between greater leisure time PA levels and lower psychological distress, while occupational PA may have detrimental effects on mental health (29). Notably, this study was the first report that greater leisure time PA may prevent the development of SI, particularly moderate or more severe SI, for military subjects. This finding was also noted in the general population (30). Additionally, our findings in patients with cardiovascular diseases following cardiac rehabilitation exercise and improvements in CRF showed reductions in psychological distress, anxiety, hostility, and especially depression as well as psychological distress-associated increased mortality (10–15).

Regular exercise regulates the activity of the hypothalamic–pituitary–adrenal axis (31), and exerts anti-inflammation effects by increasing the release of peroxisome proliferator-activated receptor gamma coactivator 1 (PGC1)-α (32). Numerous clinical and translational studies have revealed an association between psychological distress and elevated pro-inflammatory cytokines (33–35). In addition, higher PA levels elevate serotonin and norepinephrine concentrations (36), and enhance the release of neurotrophin (31). These bioactive molecules can cross the blood–brain barrier (BBB), enhance brain-derived neurotrophic factor (BDNF) signaling, and positively contribute to mental health (37–40).

This study has several limitations. First, the cross-sectional design employed in this study could not establish a causal relationship between psychological distress and PA levels. Second, the focus on military subjects in this study restricts the generalizability of the findings to the general population. Third, the PA levels in this study were assessed by running time while the effect of strength exercises on mental stress was unclear. Finally, since psychological distress involves various factors that can affect outcomes, it was not feasible to account for all potential confounding factors in this study. However, this study had some strengths. First, the living environment and medical support in the military were similar, possibly reducing the unmeasured confounding effects. Second, this study focused on leisure-time PA instead of training-related PA, which was correlated to rank in the military, and those with lower rank were likely to have higher psychological distress.

## Conclusion

Engaging in moderate-intensity PA during leisure time based on American guidelines was associated with better mental health in the military in this study. It is worth noting a dose–response phenomenon was observed in the association of greater moderate-intensity aerobic PA levels with better mental health, e.g., SI among military personnel. Further prospective longitudinal studies are needed to clarify the temporal association between PA levels and mental health.

## Data availability statement

The raw data supporting the conclusions of this article will be made available by the authors, without undue reservation.

## Ethics statement

The studies involving humans were approved by The Institutional Ethics Committee approval (No. 16-05-008) from the Mennonite Christian Hospital in Taiwan was obtained at the beginning of the study. The studies were conducted in accordance with the local legislation and institutional requirements. The participants provided their written informed consent to participate in this study.

## Author contributions

K-ZT: Data curation, Formal analysis, Investigation, Resources, Software, Visualization, Writing – original draft. P-YL: Investigation, Supervision, Visualization, Writing – review & editing. Y-PL: Investigation, Supervision, Validation, Visualization, Writing – review & editing. C-CC: Investigation, Supervision, Writing – review & editing. W-CH: Investigation, Supervision, Writing – review & editing. XS: Investigation, Supervision, Validation, Writing – review & editing. CL: Investigation, Supervision, Writing – review & editing. G-ML: Conceptualization, Data curation, Funding acquisition, Investigation, Methodology, Project administration, Resources, Supervision, Validation, Visualization, Writing – review & editing.

## References

[1] Singh B Olds T Curtis R Dumuid D Virgara R Watson A. Effectiveness of physical activity interventions for improving depression, anxiety and distress: an overview of systematic reviews. Br J Sports Med. (2023) 57:1203–9. doi: 10.1136/bjsports-2022-106195, PMID: 36796860 PMC10579187

[2] Lin GM Nagamine M Yang SN Tai YM Lin C Sato H. Machine learning based suicide ideation prediction for military personnel. IEEE J Biomed Health Inform. (2020) 24:1907–16. doi: 10.1109/JBHI.2020.2988393, PMID: 32324581

[3] Franklin JC Ribeiro JD Fox KR Bentley KH Kleiman EM Hung X. Risk factors for suicidal thoughts and behaviors: a meta-analysis of 50 years of research. Psychol Bull. (2017) 143:187–232. doi: 10.1037/bul0000084, PMID: 27841450

[4] Schoenbaum M Kessler RC Gilman SE Colpe LJ Heeringa SG Stein MB. Predictors of suicide and accident death in the Army study to assess risk and resilience in servicemembers (Army STARRS): results from the Army study to assess risk and resilience in servicemembers (Army STARRS). JAMA Psychiatry. (2014) 71:493–503. doi: 10.1001/jamapsychiatry.2013.4417 24590048 PMC4124912

[5] Zuromski KL Dempsey CL Ng THH Riggs-Donovan CA Brent DA Heeringa SG. Utilization of and barriers to treatment among suicide decedents: results from the Army study to assess risk and resilience among servicemembers (Army STARRS). J Consult Clin Psychol. (2019) 87:671–83. doi: 10.1037/ccp0000400, PMID: 31008631 PMC6625932

[6] Department of Defense (DoD) annual report on suicide in the military: Calendar year (CY) (2021).

[7] Department of Defense (DoD) quarterly suicide report (QSR) 1 st quarter, CY (2023).

[8] Bull FC Al-Ansari SS Biddle S Borodulin K Buman MP Cardon G. World health organization 2020 guidelines on physical activity and sedentary behaviour. Br J Sports Med. (2020) 54:1451–62. doi: 10.1136/bjsports-2020-102955, PMID: 33239350 PMC7719906

[9] Stubbs B Vancampfort D Hallgren M Firth J Veronese N Solmi M. EPA guidance on physical activity as a treatment for severe mental illness: a meta-review of the evidence and position statement from the European psychiatric association (EPA), supported by the International Organization of Physical Therapists in mental health (IOPTMH). Eur Psychiatry. (2018) 54:124–44. doi: 10.1016/j.eurpsy.2018.07.004, PMID: 30257806

[10] Lavie CJ Menezes AR De Schutter A Milani RV Blumenthal JA. Impact of cardiac rehabilitation and exercise training on psychological risk factors and subsequent prognosis in patients with cardiovascular disease. Can J Cardiol. (2016) 32:S365–73. doi: 10.1016/j.cjca.2016.07.508 27692117

[11] Kachur S Menezes AR De Schutter A Milani RV Lavie CJ. Significance of comorbid psychological stress and depression on outcomes after cardiac rehabilitation. Am J Med. (2016) 129:1316–21. doi: 10.1016/j.amjmed.2016.07.006 27480388

[12] Lavie CJ Sui X Milani RV. Emotional distress after myocardial infarction: importance of cardiorespiratory fitness. Eur J Prev Cardiol. (2018) 25:906–9. doi: 10.1177/2047487318770516 29651866

[13] Hughes JW Serber ER Kuhn T. Psychosocial management in cardiac rehabilitation: current practices, recommendations, and opportunities. Prog Cardiovasc Dis. (2022) 73:76–83. doi: 10.1016/j.pcad.2021.12.006 35016916

[14] Lavie CJ Zhang I Yang D Liu M. Improving fitness through exercise will improve our heart and mind. Heart Mind. (2023) 7:49–51. doi: 10.4103/hm.hm_59_22

[15] O'Keefe EL O'Keefe JH Lavie CJ. The intersection of exercise, cognition, and cardiovascular disease. Heart Mind. (2023) 7:3–4. doi: 10.4103/hm.hm_5_23

[16] Piercy KL Troiano RP Ballard RM Carlson SA Fulton JE Galuska DA. The physical activity guidelines for Americans. JAMA. (2018) 320:2020–8. doi: 10.1001/jama.2018.14854 30418471 PMC9582631

[17] Lin GM Li YH Lee CJ Shiang JC Lin KH Chen KW. Rationale and design of the cardiorespiratory fitness and hospitalization events in armed forces study in eastern Taiwan. World J Cardiol. (2016) 8:464–71. doi: 10.4330/wjc.v8.i8.464, PMID: 27621774 PMC4997527

[18] Lin KH Su FY Yang SN Liu MW Kao CC Nagamine M. Body mass index and association of psychological stress with exercise performance in military members: the cardiorespiratory fitness and hospitalization events in armed forces (CHIEF) study. Endocr Metab Immune Disord Drug Targets. (2021) 21:2213–9. doi: 10.2174/1871530321666210427090550 33906597

[19] Su FY Wang SH Lu HH Lin GM. Association of tobacco smoking with physical fitness of military males in Taiwan: the CHIEF study. Can Respir J. (2020) 2020:1–6. doi: 10.1155/2020/5968189 PMC6969999 31998426

[20] Hwang LC Bai CH Chen CJ. Prevalence of obesity and metabolic syndrome in Taiwan. J Formos Med Assoc. (2006) 105:626–35. doi: 10.1016/S0929-6646(09)60161-3 16935763

[21] Lin YK Tsai KZ Han CL Lin YP Lee JT Lin GM. Obesity phenotypes and electrocardiographic characteristics in physically active males: CHIEF study. Front Cardiovasc Med. (2021) 8:738575. doi: 10.3389/fcvm.2021.738575, PMID: 34722672 PMC8548412

[22] Wang SH Chung PS Lin YP Tsai KZ Lin SC Fan CH. Metabolically healthy obesity and physical fitness in military males in the CHIEF study. Sci Rep. (2021) 11:9088. doi: 10.1038/s41598-021-88728-0, PMID: 33907258 PMC8079407

[23] Chung PS Tsai KZ Lin YP Lin YK Lin GM. Association between leukocyte counts and physical fitness in male military members: the CHIEF study. Sci Rep. (2020) 10:6082. doi: 10.1038/s41598-020-63147-9, PMID: 32269281 PMC7142135

[24] Lee MB Liao SC Lee YJ Wu CH Tseng MC Gau SF. Development and verification of validity and reliability of a short screening instrument to identify psychiatric morbidity. J Formos Med Assoc. (2003) 102:687–94. PMID: 14691593

[25] Lin YP Fan CH Tsai KZ Lin KH Han CL Lin GM. Psychological stress and long-term blood pressure variability of military young males: the cardiorespiratory fitness and hospitalization events in armed forces study. World J Cardiol. (2020) 12:626–33. doi: 10.4330/wjc.v12.i12.626, PMID: 33391615 PMC7754384

[26] Lin KH Chen YJ Yang SN Liu MW Kao CC Nagamine M. Association of psychological stress with physical fitness in a military cohort: the CHIEF study. Mil Med. (2020) 185:e1240–6. doi: 10.1093/milmed/usz469, PMID: 32239167

[27] Wyss T Boesch M Roos L Tschopp C Frei KM Annen H. Aerobic fitness level affects cardiovascular and salivary alpha amylase responses to acute psychosocial stress. Sports Med Open. (2016) 2:33. doi: 10.1186/s40798-016-0057-9 27747788 PMC4995230

[28] Crombie KM Adams TG Dunsmoor JE Greenwood BN Smits JA Nemeroff CB. Aerobic exercise in the treatment of PTSD: an examination of preclinical and clinical laboratory findings, potential mechanisms, clinical implications, and future directions. J Anxiety Disord. (2023) 94:102680. doi: 10.1016/j.janxdis.2023.102680, PMID: 36773486 PMC10084922

[29] Martins LC Lopes CS. Rank, job stress, psychological distress and physical activity among military personnel. BMC Public Health. (2013) 13:716. doi: 10.1186/1471-2458-13-716 23914802 PMC3846587

[30] Kim K Shin YJ Nam JH Choi BY Kim MK. A dose-response relationship between types of physical activity and distress. J Korean Med Sci. (2008) 23:218–25. doi: 10.3346/jkms.2008.23.2.218 18437003 PMC2526420

[31] Lopresti AL Hood SD Drummond PD. A review of lifestyle factors that contribute to important pathways associated with major depression: diet, sleep and exercise. J Affect Disord. (2013) 148:12–27. doi: 10.1016/j.jad.2013.01.014 23415826

[32] Handschin C Spiegelman BM. The role of exercise and PGC1alpha in inflammation and chronic disease. Nature. (2008) 454:463–9. doi: 10.1038/nature07206, PMID: 18650917 PMC2587487

[33] Raison CL Capuron L Miller AH. Cytokines sing the blues: inflammation and the pathogenesis of depression. Trends Immunol. (2006) 27:24–31. doi: 10.1016/j.it.2005.11.006, PMID: 16316783 PMC3392963

[34] Weber MD Godbout JP Sheridan JF. Repeated social defeat, neuroinflammation, and behavior: monocytes carry the signal. Neuropsychopharmacology. (2017) 42:46–61. doi: 10.1038/npp.2016.102, PMID: 27319971 PMC5143478

[35] Nobis A Zalewski D Waszkiewicz N. Peripheral markers of depression. J Clin Med. (2020) 9:3793. doi: 10.3390/jcm9123793, PMID: 33255237 PMC7760788

[36] Ross RE VanDerwerker CJ Saladin ME Gregory CM. The role of exercise in the treatment of depression: biological underpinnings and clinical outcomes. Mol Psychiatry. (2023) 28:298–328. doi: 10.1038/s41380-022-01819-w 36253441 PMC9969795

[37] Khoury R Nagy C. Running from stress: a perspective on the potential benefits of exercise-induced small extracellular vesicles for individuals with major depressive disorder. Front Mol Biosci. (2023) 10:1154872. doi: 10.3389/fmolb.2023.1154872 37398548 PMC10309045

[38] Sleiman SF Henry J Al-Haddad R Hayek LE Haidar EA Stringer T. Exercise promotes the expression of brain derived neurotrophic factor (BDNF) through the action of the ketone body beta-hydroxybutyrate. elife. (2016) 5:e15092. doi: 10.7554/eLife.15092, PMID: 27253067 PMC4915811

[39] Karnib N El-Ghandour R El Hayek L Nasrallah P Khalifeh M Barmo N. Lactate is an antidepressant that mediates resilience to stress by modulating the hippocampal levels and activity of histone deacetylases. Neuropsychopharmacology. (2019) 44:1152–62. doi: 10.1038/s41386-019-0313-z, PMID: 30647450 PMC6461925

[40] Russo-Neustadt AA Beard RC Huang YM Cotman CW. Physical activity and antidepressant treatment potentiate the expression of specific brain-derived neurotrophic factor transcripts in the rat hippocampus. Neuroscience. (2000) 101:305–12. doi: 10.1016/S0306-4522(00)00349-3, PMID: 11074154

