# Supplementary material for: Do the American guideline-based leisure time physical activity levels for civilians benefit the mental health of military personnel?
Source: Front Psychiatry. 2023 Nov 14;14:1255516. doi: 10.3389/fpsyt.2023.1255516 (PMC10682789; doi:10.3389/fpsyt.2023.1255516)
Supplement: Supplementary file 1 [file Table_1.docx]

**Supplementary Table 1.** Association of physical activity levels with different grades of psychological distress

| PA levels | Model 1 (Unadjusted) | | | | | | | |
| --- | --- | --- | --- | --- | --- | --- | --- | --- |
| (min/wk) | BSRS-5 ≥15  (n = 11) | |  | BSRS ≥10  (n = 91) | |  | BSRS >5  (n = 385) | |
|  | OR (95% CI) | *P* value |  | OR (95% CI) | *P* value |  | OR (95% CI) | *P* value |
| <150 | 1.00 |  |  | 1.00 |  |  | 1.00 |  |
| 150-299 | 0.29 (0.07 – 1.15) | 0.07 |  | 0.83 (0.51 – 1.34) | 0.44 |  | 0.83 (0.65 – 1.07) | 0.14 |
| ≥300 | 0.18 (0.04 – 0.90) | 0.03 |  | 0.37 (0.21 – 0.66) | 0.001 |  | 0.40 (0.30 – 0.53) | <0.001 |
|  |  |  |  |  |  |  |  |  |
|  | Model 2 (Multivariable-adjusted)* | | | | | | | |
| <150 | 1.00 |  |  | 1.00 |  |  | 1.00 |  |
| 150-299 | 0.29 (0.07 – 1.19) | 0.08 |  | 0.83 (0.51 – 1.35) | 0.45 |  | 0.80 (0.62 – 1.03) | 0.08 |
| ≥300 | 0.21 (0.04 – 1.04) | 0.056 |  | 0.38 (0.21 – 0.69) | 0.001 |  | 0.41 (0.31 – 0.55) | <0.001 |

Abbreviations: BSRS. Brief Symptom Rating Scale; PA, physical activity; OR, odds ratio; CI, confidence interval.

*adjusted for age, sex, specialty, cigarette smoking, alcohol drinking, betel nut chewing, general obesity, and abdominal obesity.
